# *Clonorchis sinensis* granulin promotes malignant transformation of human intrahepatic biliary epithelial cells through interaction with M2 macrophages via regulation of STAT3  phosphorylation and the MEK/ERK pathway

**DOI:** 10.1186/s13071-023-05765-6

**Published:** 2023-04-24

**Authors:** Qing He, Xiaowen Pan, Yingxuan Yin, Anyuan Xu, Xueqing Yi, Yinjuan Wu, Xuerong Li

**Affiliations:** 1 Department of Parasitology, Zhongshan School of Medicine, Sun Yat-Sen University, Guangzhou, Guangdong 510080 People’s Republic of China; 2 Key Laboratory for Tropical Diseases Control of the Ministry of Education, Sun Yat-Sen University, Guangzhou, Guangdong 510080 People’s Republic of China; 3 Provincial Engineering Technology Research Center for Biological Vector Control, Guangzhou, Guangdong 510080 People’s Republic of China; 4 China Atomic Energy Authority Center of Excellence on Nuclear Technology Applications for Insect Control, Beijing, 100048 People’s Republic of China; 5 Department of Basic Medicine, Zhongshan School of Medicine, Sun Yat-Sen University, Guangzhou, Guangdong 510080 People’s Republic of China

**Keywords:** *Clonorchis sinensis* granulin, Malignant transformation, M2 macrophages, STAT3 phosphorylation, MEK/ERK signaling pathway

## Abstract

**Background:**

*Clonorchis sinensis* granulin (*Cs*GRN), a component of the excretory/secretory products of this species, is a multifunctional growth factor that can promote the metastasis of cholangiocarcinoma cells. However, the effect of *Cs*GRN on human intrahepatic biliary epithelial cells (HIBECs) is unclear. Here, we investigated the effect of *Cs*GRN on the malignant transformation of HIBECs and its possible underlying mechanism.

**Methods:**

The malignant transformation phenotypes of HIBECs after *Cs*GRN treatment were estimated by EdU-488 incorporation assay, colony formation assay, wound-healing assay, Transwell assay and western blot. The biliary damage of *Cs*GRN-treated mice was detected by western blot, immunohistochemical staining and hematoxylin and eosin staining. The phenotypes of the macrophages [human monocytic leukemia cell line (THP-1)] were analyzed by flow cytometry, immunofluorescence and immunohistochemistry, both in vitro and in vivo. A co-culture system was developed to explore the interaction between THP-1 and HIBECs in *Cs*GRN-containing medium. Enzyme-linked immunosorbent assay and western blot were used to detected the activation of interleukin 6 (IL-6), phosphorylated signal transducer and activator of transcription 3 (p-STAT3) and the mitogen-activated protein kinase (MEK)/extracellular signal-regulated kinase (ERK) pathway. An inhibitor of the MEK/ERK pathway, PD98059, was used to determine whether this pathway is involved in *Cs*GRN-mediated cell interactions as well as in STAT3 phosphorylation and malignant transformation of HIBECs.

**Results:**

Excessive hyperplasia and abnormal proliferation of HIBECs, enhanced secretion of hepatic pro-inflammatory cytokines and chemokines, as well as biliary damage, were observed in vitro and in vivo after treatment with *Cs*GRN. The expression of the markers of M2 macrophages significantly increased in *Cs*GRN-treated THP-1 cells and biliary duct tissues compared with the controls. Moreover, following treatment with *Cs*GRN, the HIBECs underwent malignant transformation in the THP-1-HIBECs co-culture group. In addition, high expression of IL-6 was observed in the *Cs*GRN-treated co-culture media, which activated the phosphorylation of STAT3, JAK2, MEK and ERK. However, treatment with an inhibitor of the MEK/ERK pathway, PD98059, decreased expression of p-STAT3 in *Cs*GRN-treated HIBECs and further repressed the malignant transformation of HIBECs.

**Conclusions:**

Our results demonstrated that, by inducing the M2-type polarization of macrophages and activating the IL-6/JAK2/STAT3 and MEK/ERK pathways in HIBECs, *Cs*GRN promoted the malignant transformation of the latter.

**Graphical abstract:**

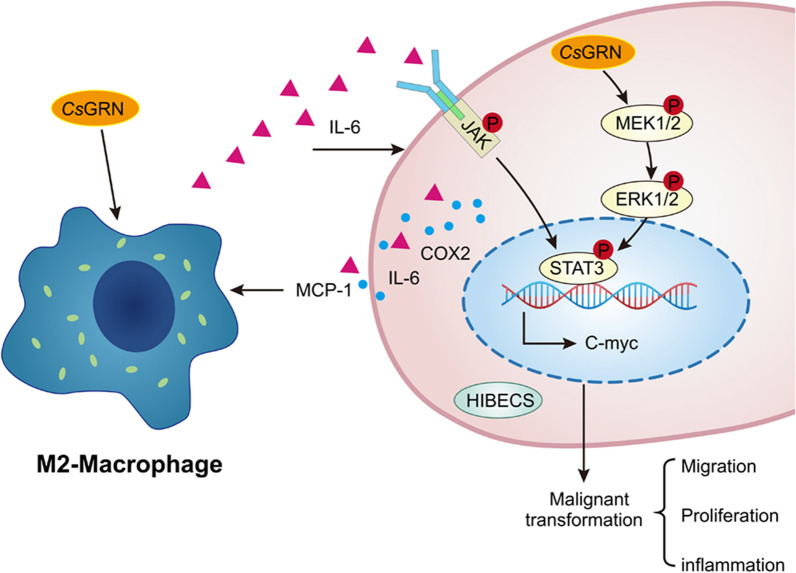

**Supplementary Information:**

The online version contains supplementary material available at 10.1186/s13071-023-05765-6.

## Background

Long-lasting infections with the food-borne parasite *Clonorchis sinensis*, the Chinese or oriental liver fluke, can result in severe symptoms and serious illness in humans. Over 15 million people are infected by *C. sinensis* worldwide, mostly in China, Korea, and Vietnam [1]. In humans, the metacercariae of *Clonorchis sinensis *excyst in the duodenum and then migrate to the bile ducts to further develop into adults [2]. Adult *C. sinensis* can survive in bile ducts for up to 30 years, where they can cause bile duct hyperplasia, periductal fibrosis, cirrhosis, and even trigger cholangiocarcinoma (CCA) [3]. The prognosis is usually dismal due to the long lifespan of *C. sinensis*, its high invasion capacity, association with early metastasis, and the low sensitivity to anticancer drugs of the latter [4–6]. Although several studies published in recent decades revealed that *C. sinensis* infection is closely associated with CCA [1, 7–11], the underlying mechanisms of this have yet to be fully elucidated.

Excretory-secretory products of *C. sinensis* (*Cs*ESPs) comprise a variety of soluble proteins and other factors that mediate many aspects of the interaction between humans and the parasite, such as digestion of nutrients, tissue invasion, cell proliferation, and regulation of the host immune system [12]. According to the results of our previous studies [13, 14], *C. sinensis* granulin (*Cs*GRN) is one of the most important components of *Cs*ESPs; it can promote the metastasis of CCA and hepatocellular carcinoma (HCC) and induce the malignant transformation of hepatocytes in vitro. Over the long-term, *Cs*ESPs can cause chronic irritation and prolonged inflammation, which is strongly associated with carcinogenesis. Macrophages play key roles in the initiation, maintenance, and resolution of inflammation. It is well-known that macrophages are capable of displaying different and even opposing phenotypes, depending on their microenvironment. Activated macrophages are usually categorized into M1 (classically activated macrophages) and M2 (alternatively activated macrophages) phenotypes. In general, M1 macrophages are regarded as anti-tumor, while M2 polarized macrophages tend to prompt many pro-tumorigenic outcomes in cancer through angiogenic, immune suppression, tumor cell proliferation, and metastasis [15]. It has been reported that M2-type macrophages can influence the migration and growth of hepatoma cells by secreting hepatocyte growth factor [16]. The number of M1 hepatic macrophages was shown to increase in early stages of *C. sinensis* infection, but there was a gradual switch to M2 hepatic macrophages during late stages, particularly during fibrosis and cirrhosis [17]. However, the role of macrophages that have undergone dynamic polarization in the *Cs*GRN-induced malignant transformation of intrahepatic biliary epithelial cells is still unclear.

In this study, we investigated the possible molecular mechanisms of *Cs*GRN in the interactions between human intrahepatic biliary epithelial cells (HIBECs) and macrophages in the malignant transformation of HIBECs. We found that *Cs*GRN initiated an inflammatory response in biliary cells and thus promoted the malignant transformation of HIBECs. In addition, the macrophages secreted a large amount of interleukin -6(IL-6), which is required for the facilitation of the abnormal proliferation of HIBECs by *Cs*GRN. IL-6 further activated the Janus kinase (JAK)/signal transducer and activator of transcription 3 (STAT3) and mitogen-activated protein kinase (MEK)/extracellular signal-regulated kinase (ERK) pathway in HIBECs, which lead to the abnormal proliferation and migration of HIBECs. Our study provides a better understanding of the function of macrophages and the interactions that occur between cells during the development of *Cs*GRN-induced malignant transformation of HIBECs, and may thus be useful for the development of novel therapeutic strategies for cholangiopathies induced by *C. sinensis* infection.

## Methods

### Cell culture and reagents

HIBEC lines and a human monocytic leukemia cell line (THP-1) were obtained from the Center of Hepato-Pancreato-Biliary Surgery in the First Affiliated Hospital of Sun Yat-sen University. Both HIBECs and THP-1 cells were cultured in RPMI-1640 medium (Gibco, Carlsbad, USA) supplemented with 10% fetal bovine serum (Gibco, USA) with 5% CO_2_ at 37 °C. THP-1 cells were treated with 200 ng/mL of phorbol-12-myristate-13-acetate (PMA) (HY-18739; MedChemExpress, USA) for 48 h and then treated with 20 ng/mL of IL-4 (NBP2–35131; Novus, USA) to generate M2 polarization or treated with 100 ng/mL of lipopolysaccharide (HY-D1056; MedChemExpress) to generate M1 polarization.

Recombinant Maltose Binding Protein (MBP) and MBP-*Cs*GRN were obtained from our previous study [14]. The *Cs*GRN fragment was amplified using forward primers 5′-ATA AGG ATC CCG GAG CAC AGGTGTAG-3′ (EcoRI) and reverse primers 5′-CGC GGA TCC TGT AAA TATA ACC AGA CTT G-3′ (BamHI), under the following conditions: 30 s at 94 °C for denaturation, 30 s at 60 °C for annealing, and 1 min at 72 °C for extension for 30 cycles. *Cs*GRN was cloned into the protocadherin (pCDH)-luc vector plasmid (identifier, CS-HLUC-pCDH; GeneCopoeia, China) to construct the recombinant plasmid pCDH-luc-*Cs*GRN. The recombinant plasmids were purified using the endotoxin-free Maxiprep Kit (Qiagen, USA) and then transformed into *Escherichia coli* DH5α for amplification (Promega, USA). Finally, the recombinant plasmid was then extracted with E.Z.N.A. Plasmid Mini Kit I (Omega, USA).

### Animal studies

BALB/c wild-type mice aged 6–8 weeks were purchased from the Laboratory Animal Center of Sun Yat-sen University. The animal use protocol was reviewed and approved by the Institutional Animal Care and Use Committee (IACUC), Sun Yat-sen University (no. SYSU-IACUC-2022-000370). The protocol was as follows: each mouse was intravenously injected with 2 ml saline dissolved in 20 μg pCDH-luc vector or 20 μgpCDH-luc-*Cs*GRN plasmid once every week, a total of four times, respectively. The plasmid solution was rapidly injected, i.e. within 8 s, through the lateral tail vein into five mice per group. The pCDH-luc vector was used as the control. The mice were checked once a week to determine whether they were suffering from abdominal distension or other health problems. Four weeks after injection, all of the mice were sacrificed with anaesthetic at the end of the experiment. The serum and proteins from the liver tissues were extracted for the following analyses.

### Detection of the location of pCDH-luc-*Cs*GRN in mice

The location of the injected *Cs*GRN plasmid in the mice was detected by an in vivo imaging system (IVIS 100; Xenogen, Alameda, CA). Briefly, 4 weeks after injection of the pCDH-luc vector or pCDH-luc-*Cs*GRN, the animals were anaesthetized. After treatment with 100 μl of D-luciferin (HY-12591A; MedChemExpress) solution (10 mg/ml) through intraperitoneal injection, the *Cs*GRN plasmid was observed by fluorescence imaging and analyzed by IVIS 100.

### Establishment of the co-culture system

Transwell chambers (0.4/8 μm PET; Millipore) were used for cell co-cultivation. After preconditioning the HIBECs for 24 h with 10 μg/ml of MBP-*Cs*GRN, the supernatant was collected and used to create the co-culture system with THP-1 cells. HIBECs were cultured in the lower chambers. THP-1 cells were cultured in the upper chambers. The THP-1 cells treated with 10 μg/ml MBP-*Cs*GRN are henceforth referred to as the THP-1-MBP-*Cs*GRN group. The THP-1 cells treated with the supernatant of HIBECs in the MBP-*Cs*GRN group are henceforth referred to as the THP-1-HIBEC-MBP-*Cs*GRN group. The THP-1 cells treated with 200 ng/ml of PMA for 48 h and 20 ng/ml of IL-4 for 48 h are henceforth referred to as the THP-1-PMA/IL-4 group.

### Measurement of cell proliferation

Cell proliferation was estimated by employing the EdU-488 incorporation assay kit (C0071S; Beyotime, China) and a colony formation assay. The treated cells were labeled with EdU-488 for 2 h following the product protocol. 4′,6-Diamidino-2-phenylindole (DAPI) was used to stain the nuclei, for 10 min. The number of cells with EdU-488 incorporation was observed by fluorescence microscopy (Leica DMI8; Leica, Wetzlar, Germany) and analyzed by ImageJ software (National Institutes of Health, USA). The colony formation assay was used to analyze the long-term effects of *Cs*GRN on HIBEC proliferation. Briefly, 1000 cells were cultivated in 24-well plates and treated with 10 μg/ml of recombinant MBP-*Cs*GRN or MBP for 14 days. The fixed cells were then stained by crystal violet (Sigma-Aldrich, St. Louis, USA) after 30 min in 4% paraformaldehyde. After washing with double-distilled H_2_O three times, the colony numbers were determined by ImageJ software (NIH).

### Measurement of cell migration

Cell migration was detected by a wound-healing assay and Transwell assay. For the cell migration effect of *Cs*GRN on HIBECs, the latter were inoculated into 6-well plates at 80% density and wounds were created using 10-L plastic pipettes. Then the cells were treated with 10 μg/ml of recombinant MBP-*Cs*GRN or MBP for 24 h, followed by observation and imaging by light microscopy (Leica DMI3000B; Leica). Treated HIBEC (5.0 × 10^4^ ) were cultured in 24-well Transwell upper chambers (Costar, New York) for 24 h, while medium containing 10% fetal bovine serum was added to the lower chambers. The migrant cells were then stained with 10% crystal violet (Sigma-Aldrich) for 10 min and counted under a light microscope (Leica DMI3000B; Leica).

### Histopathology

The mouse livers were frozen in 10% formalin for 24 h at room temperature, dehydrated and transparentized, then embedded in paraffin and sectioned into 5-μm-thick slices. Hematoxylin and eosin staining was followed by immunohistochemical (IHC)/immunofluorescence (IF) analyses of the sections.

### Immunohistochemistry

Tissue samples were sectioned into 5-μm-thick slices and incubated with 3% H_2_O_2_ at room temperature for 10 min, then blocked with 3% bovine serum albumin (BSA) at 37 °C for 30 min. The tissues were then incubated with primary antibodies at 4 °C overnight and then with secondary antibodies at room temperature for 1 h. The following primary antibodies were purchased from Cell Signaling Technology (CST, Boston, USA) and Abcam Shanghai Trading Corporation (Abcam, Shanghai, China): cytokeratin 19 (CK19), MCP-1, CD206 and INOS. The HRP-conjugated secondary antibodies were purchased from Proteintech Group, USA. The immunohistochemical staining was imaged using Caseviewer software (3DHISTECH, Hungary).

### IF assay

The liver tissue specimens were fixed in 4% paraformaldehyde at room temperature for 10 min, permeabilized with 0.1% Triton X-100 (9002–93-1; Beijing Biotopped Science & Technology, China) for 10 min, and then incubated with 3% BSA in PBS at room temperature for 30 min. The tissues were then incubated with primary antibodies [CD68, 1:100; CD163, 1:100, CK19, 1:100; phosphorylated (p-) STAT3 (p-STAT3), 1:200; p-JAK2, 1:100; p-ERK, 1:100; p-MEK, 1:100; Abcam, Cambridge, UK] at 4 °C overnight and with secondary antibodies [Alexa Fluor 488 conjugated goat anti-rabbit immunoglobulin G (IgG); Alexa Fluor 594 conjugated goat anti-mouse IgG (Invitrogen, Waltham, USA)] at room temperature for 1 h. The nuclei were stained with DAPI (Abcam, Cambridge, USA) for 10 min at room temperature. The liver tissue specimens of each animal were imaged six times using confocal laser scanning microscopy (LSM780; Zeiss, Oberkochen, Germany). For each mouse liver, the average signal value of six images is presented as the real value.

### Western blot and enzyme-linked immunosorbent assay 

After cells or liver homogenates had been harvested and washed in cold PBS twice, proteins were extracted using a radioimmunoprecipitation assay solution (Beyotime, Shanghai, China), and the protein concentration was measured using a bicinchoninic acid assay protein assay kit (Thermo, Shanghai, China). Western blot was undertaken in accordance with a previous study [14]. All primary antibodies—anti-E-cadherin (1:2000), anti-vimentin (1:2,000), anti-N-cadherin (1:2000), anti-tight junction protein (ZO-1) (1:2000), anti-β-catenin (1:2000), anti-IL6 (1:2000), anti-cyclooxygenase-2 (COX-2) (1:2000), anti-MCP-1 (1:2000), anti-p-JAK2 (1:2000), anti-, p-STAT3 (1:2000), anti-c-Myc (1:2000), anti-p-MEK (1:2000), anti-p-ERK (1:2,000), anti-STAT3 (1:2000), anti-MEK (1:2000), anti-ERK (1:2000) and anti-GAPDH (1:2000) were purchased from Cell Signaling Technology (Boston, USA). Enzyme-linked immunosorbent assay (ELISA) kits (Multi Sciences, Hangzhou, China) were used to determined IL-6 and IL-10 levels in THP-1 cells and the co-culture medium.

### Flow cytometry

THP-1 cells and hepatic macrophages in each group were harvested and stained with APC/CY7 anti-mouse CD45 (103116; BioLegend, USA), PE anti-mouse CD11b (101208; BioLegend), APC anti-mouse F480 (123116; BioLegend), PC7 anti-mouse CD206 (141720; BioLegend), PC7 anti-human CD206 (T7-782-T100, EXBIO) and PC5.5 anti-mouse MHC-II (107626; BioLegend) antibodies at 4 °C for 30 min. Hepatic macrophages were detected in the mice by grinding the liver tissues gently, filtering the homogenate through a 80-μm nylon mesh filter, and isolating hepatic mononuclear cells with 40% and 80% Percoll (17-0891-01; GE Healthcare, UK). The isolated cells were stained with the above surface markers after being blocked with anti-mouse CD16/32 (TruStain FcX PLUS; BioLegend) following red blood cell lysis (420301; BioLegend, USA). After staining, the cells were washed three times with PBS and resuspended with 200 μl of 10% BSA diluted with PBS, and analyzed by FACS with a BD FACS Aria II (BD Science, USA). The data were analyzed using FlowJo v10 (TreeStar, Ashland, USA).

### Quantitative polymerase chain reaction

Total RNA from the liver homogenates was extracted using TRIzol solution, according to the manufacturer’s protocol (Invitrogen, Carlsbad, USA). A quantitative polymerase chain reaction (qPCR) kit (Vazyme, Nanjing, China) was used to quantify the gene expression level. The PCR conditions were as in a previous study [13]. The primer sequences were as follows: *Cs*GRN, F-5′-CGC GGA TCC TGT AAA TAT AAC CAG ACT TG-3′, R-5′-TTA CTC GAG CGG AGC ACA GGT GTA GTG AT-3′; iNOS, F-5′-GCA CAG GAA ATG TTC ACC TAC-3′, R-5′-CAC GAT GGT GAC TTT GGC TAG-3′; Arg1, F-5′-ACG GAA GAA TCA GCC TGG TG-3′, R-5′-GTC CAC GTC TCT CAA GCC AA-3′; STAT3, F-5′-CAG CAG CTT GAC ACA CGG TA-3′, R-5′-AAA CAC CAA AGT GGC ATG TGA-3′; Bcl-2, F-5′-GGT GGG GTC ATG TGT GTG G-3′, R-5′-C GGT TCA GGT ACT CAG TCA TCC-3′; TFF3, F-5′-CCA AGC AAA CAA TCC AGA GCA-3′, R-5′-GCT CAG GAC TCG CTT CAT GG-3′; c-Myc, F-5′-GTC AGT TCG GGA AGG CTG TA-3′, R-5’-AAT CGG AGT TGG AAT CAG TCA C-3′; GAPDH, F-5′-ACG ACC ACT TTG TCA AGC TC-3′, R-5′-GTG AGG AGG GGA GAT TCA GT-3′.

### Statistical analysis

All the results are from three independent experiments and are presented as means ± SD. GraphPad Prism 8.0 software (San Diego, CA) was used for statistical analysis. Analyses of statistical differences were conducted using Student's* t*-test and ANOVA*.*

## Results

### *Cs*GRN induced the malignant transformation of HIBECs

**Fig. 1:**
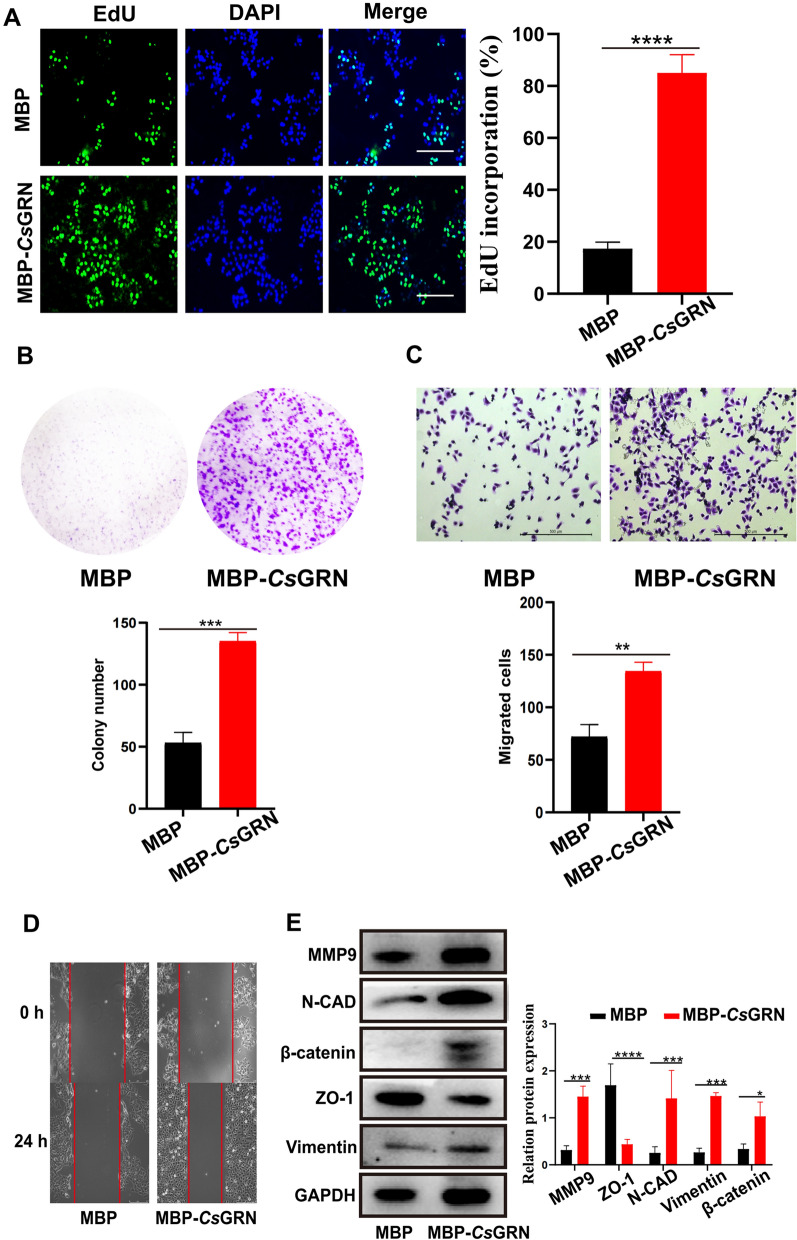
**a**–**e** *Clonorchis sinensis* granulin (*CsGRN*) induces the malignant proliferation, metastasis and epithelial-to-mesenchymal transition (EMT) of human intrahepatic biliary epithelial cells (HIBECs)*.* An EdU-488 assay (**a**) and colony formation assay (**b**) were conducted to determine the proliferation capability of HIBEC cells treated with 10 μg/ml of recombinant Maltose Binding Protein (MBP)-*Cs*GRN or MBP after 24 h, respectively. Scale bar 10 μM. A Transwell assay (**c**) and wound healing assay (**d**) were performed to estimate the invasion capability of HIBECs treated with 10 μg/ml MBP-*Cs*GRN or MBP after 24 h. **e** Western blot was used to estimate the expression level of EMT-related proteins. * *P* < 0.05, ** *P* < 0.01, *** P < 0.001, **** *P* < 0.0001. *DAPI* 4′,6-Diamidino-2-phenylindole,* MMP9* matrix metalloproteinase 9,* N-CAD* N-cadherin,* ZO-1* tight junction protein, *GADPH* glyceraldehyde 3-phosphate dehydrogenase

We previously found that co-culture of *Cs*GRN can promote the metastasis of CCA and HCC and induce the malignant transformation of hepatocytes [13, 14]. However, the effect of *Cs*GRN on HIBECs remains unclear. Here, we used 10 μg/ml of MBP-*Cs*GRN to treat HIBECs, and found that recombinant *Cs*GRN protein treated-HIBECs grew quickly and formed more cell clones compared with the control (Fig. 1a, b), indicating that *Cs*GRN can promote the abnormal proliferation of bile duct epithelial cells in vitro. Moreover, we also observed significantly increased migration activity of HIBECs after their treatment with 10 μg/ml of MBP-*Cs*GRN for 24 h (Fig. 1c, d). As epithelial-to-mesenchymal transition (EMT) plays a critical role in carcinogenesis [18], especially in the process of migration and invasion, we further investigated if* Cs*GRN caused EMT in HIBECs. Several biomarkers of EMT were determined by western blotting. As shown in Fig. 1e, when co-cultured with recombinant *Cs*GRN protein, there was a significant increase in matrix metalloproteinase 9, N-cadherin, vimentin, and β-catenin, and decrease in ZO-1, in HIBEC cells compared with the control, which indicates that *Cs*GRN can induce EMT in HIBECs. Collectively, these results suggest that *Cs*GRN can promote the malignant transformation of normal HIBECs.

### *Cs*GRN caused bile duct injury through upregulating the production of pro-inflammatory cytokines and chemokines

**Fig. 2:**
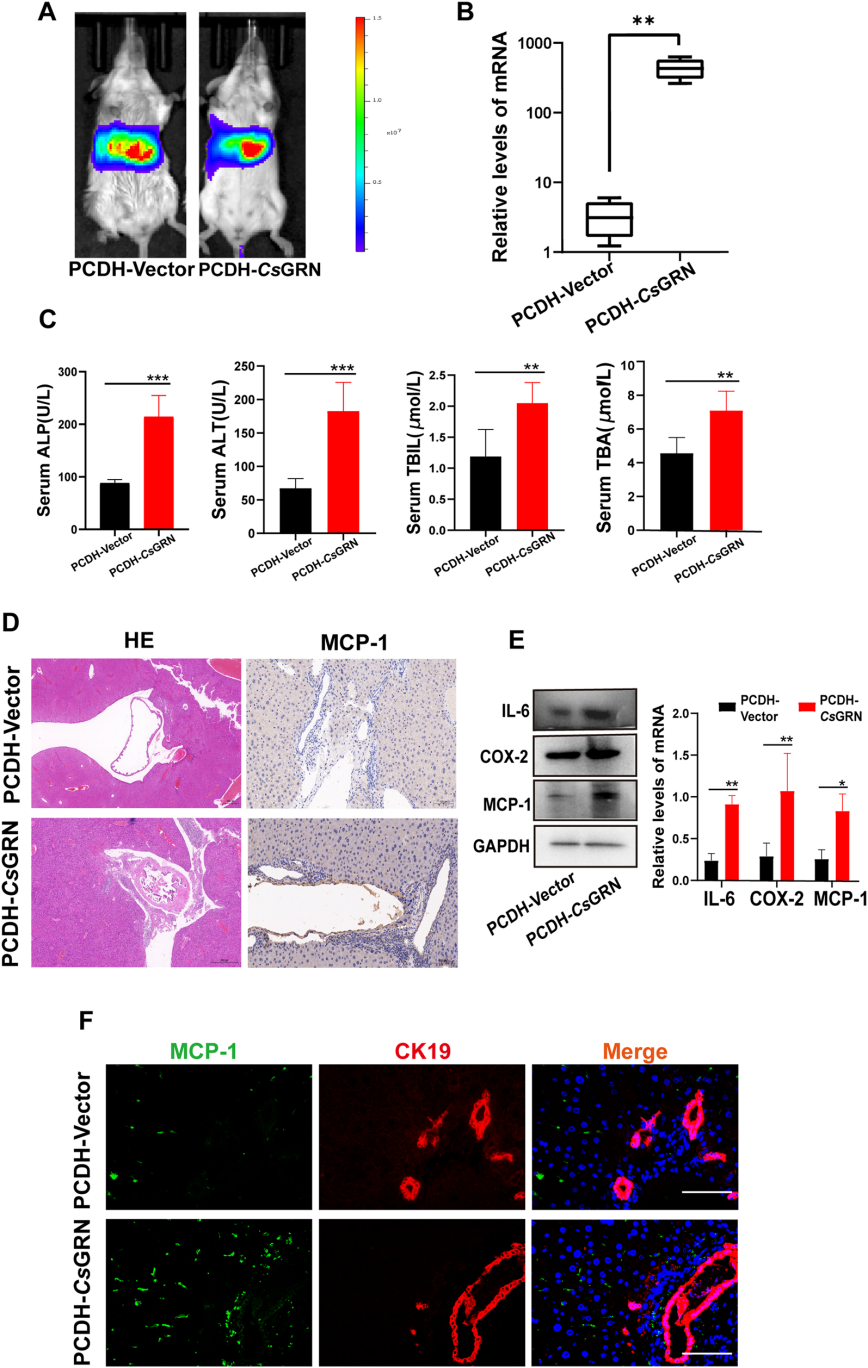
**a**–**f** *Cs*GRN induces biliary damage and inflammatory cell infiltration both in vitro and in vivo. **a** The location of injected recombinant plasmid in mice was observed through an in vivo imaging system (IVIS). The color scale indicates the fluorescence intensity of the firefly luciferase gene attached to the plasmid. **b** Messenger RNA (*mRNA*) expression levels of *Cs*GRN in liver tissue were measured by quantitative polymerase chain reaction (qPCR). Data are shown as a standard boxplot. **c** The levels of serum indicators for biliary damage, such as alkaline phosphatase (*ALP*), alanine aminotransferase (*ALT*), total bilirubin (*TBIL*), and total bile acid (*TBA*), in the mice treated by *pCDH*-luc vector or pCDH-luc-*Cs*GRN were measured by enzyme-linked immunosorbent assay (ELISA). **d** Hematoxylin and eosin (*HE*) staining was used to observe the histological changes in the bile ducts of mice treated by pCDH-luc vector or pCDH-luc-*Cs*GRN (left panels). Scale bar 500 μM. Immunohistochemical (IHC) staining was used to detect the expression of MCP-1 (right panels). Scale bar 100 μM. **e** Western blot was used to detect the expression of MCP-1, interleukin-6 (*IL-6*), and cyclooxygenase-2 (*COX-2*) proteins in the liver. **f** Immunofluorescence (IF) after double staining with cytokeratin 19 (*CK19*) and MCP-1 in the portal area of *Cs*GRN-treated mice livers. MCP-1 (green), CK19 (red), DAPI (blue). Scale bar 10 μM. * *P* < 0.05, ** *P* < 0.01, *** P < 0.001. For other abbreviations, see Fig. 1

Abnormal inflammation can cause tissue injury which, together with wound repair, can play a leading role in oncogenesis. To identify the inflammation phase in hosts due to *Cs*GRN, we intravenously injected recombinant plasmid (pCDH-Luc-*Cs*GRN/pCDH vector) into mice. We observed that recombinant plasmids were enriched in the liver (Fig. 2a), and mice injected with pCDH-Luc-*Cs*GRN had a higher expression level of *Cs*GRN (Fig. 2b). We also found that biliary injury biomarkers, such as alkaline phosphatase, alanine aminotransferase, total bilirubin, and total bile acid, were markedly elevated in serum (Fig. 2c). Hematoxylin and eosin staining indicated that *Cs*GRN altered biliary structures and induced massive inflammation (Fig. 2d). We then estimated the expression levels of pro-inflammatory cytokines and chemokines in the liver. Western blot revealed that *Cs*GRN robustly increased the expression of MCP-1, IL-6 and COX-2, both in vitro (Additional file 1: Figure S1A) and in vivo (Fig. 2e). IHC staining and the IF results also revealed an increase in MCP-1 in the injured biliary tissue after *Cs*GRN treatment (Fig. 2d, f).

### *Cs*GRN induced M2-like macrophage activation

**Fig. 3:**
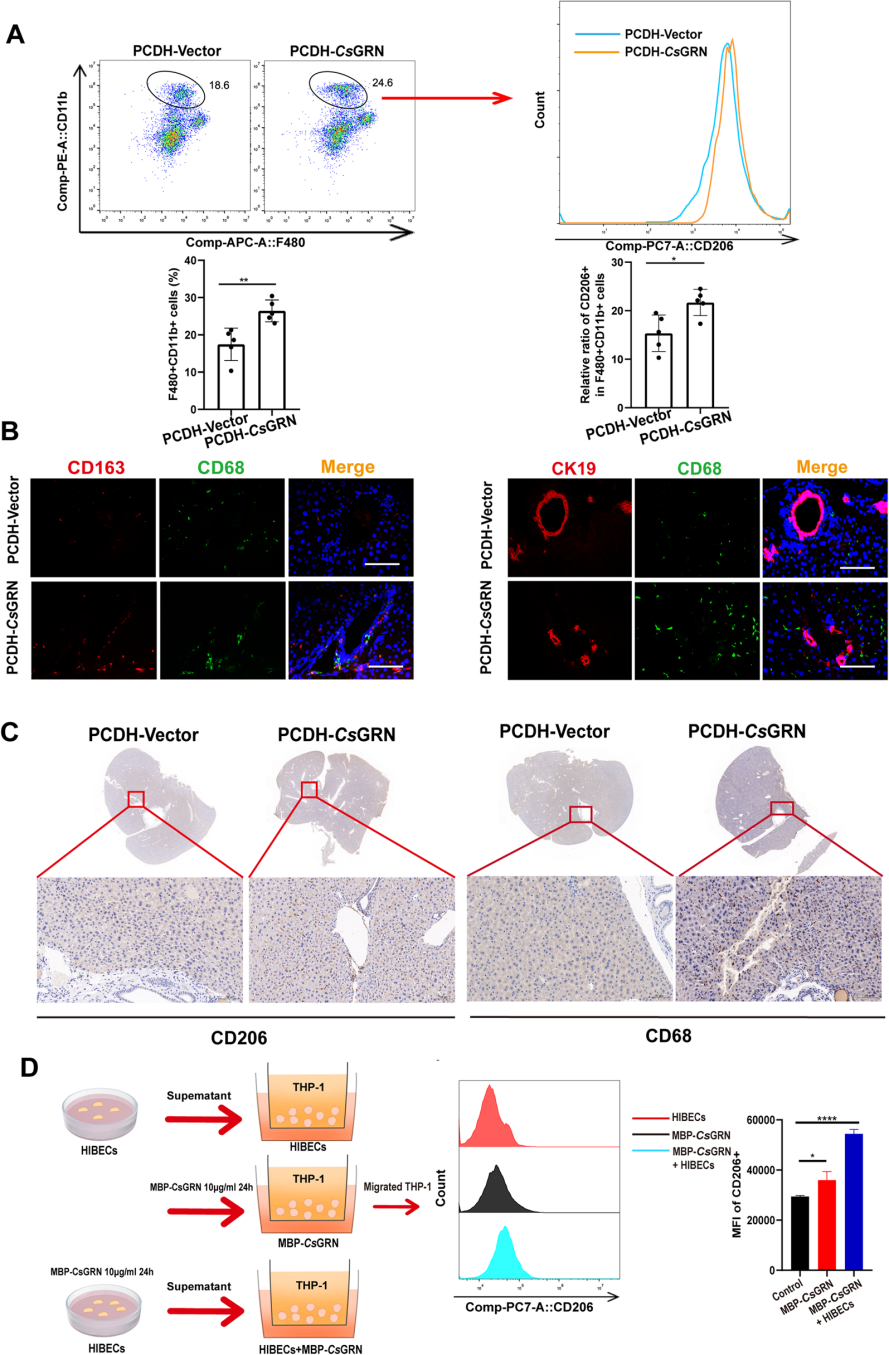
**a**–**d** M2 macrophages are activated by *Cs*GRN and infiltration if predominantly in the portal area of *Cs*GRN-treated mice. **a** Flow cytometry was used to measure the expression of CD48+ CD11b+ cells and CD480+ CD11b+ CD206+ cells in the mice livers*.* **b** Proportion of CD68+ CD163+ macrophages (M2-like) and CK19+ CD68+ macrophages in the livers of mice were determined using an IF assay. CD68 (green), CD163/CK19 (red), DAPI (blue). Scale bar 10 μM. **c** Expression of CD206 and CD68 in liver tissue was measured by an IHC assay. Scale bar 100 μM.** d** The expression of CD206+ cells of migrated human monocytic leukemia cell line (*THP-1*) cells was detected by flow cytometry. * *P* < 0.05, ** *P* < 0.01, **** P < 0.0001. For other abbreviations, see Figs. 1 and 2

Since MCP-1 is able to convert monocytes to macrophages [19], and macrophages play a critical role in the regulation of biliary function and hepatic homeostasis, we investigated the biological role of *Cs*GRN with respect to macrophages. We first determined the effects of *Cs*GRN on the regulation of hepatic macrophages in mice. Compared with the control groups of mice, *Cs*GRN-treated groups had higher expression of F480+, CD11b+ cells (macrophage marker), and F480+ CD11b+ CD206+ cells (M2 marker) in the liver tissue (Fig. 3a). Moreover, the IF analyses revealed that M2 macrophages (CD68+ , CD163+) and activated macrophages (CK19+ , CD68+) were enriched around biliary ducts in *Cs*GRN-treated groups (Fig. 3b). In addition, IHC staining revealed an increase in CD206+ and CD68+ in the injured biliary tissue after *Cs*GRN treatment (Fig. 3c). We established a co-culture system (Fig. 3d) to investigate if monocytes showed chemotaxis towards HIBECs treated with MBP-*Cs*GRN proteins. THP-1 cells were co-cultured with MBP-*Cs*GRN proteins or cell culture supernatant from HIBECs pre-treated with MBP-*Cs*GRN proteins for 24 h. HIBECs without MBP-*Cs*GRNs were used as the control. HIBECs pre-treated with MBP-*Cs*GRN significantly increased the migration activity of THP-1 cells (Additional file 1: Figure S1B). The expression of CD206+ of migrated THP-1 cells was detected by flow cytometry. The combination of MBP-*Cs*GRN and HIBECs up-regulated the expression of CD206+ cells in M2-type macrophages (Fig. 3d). Moreover, qPCR and ELISA showed that the expression of Arg1 (M2-related molecule) and IL-10 (M2-related cytokine) in migrated THP-1 cells treated with *Cs*GRN and HIBECs was also significantly increased, while the expression of iNOS (M1-related molecule) and IL-6 (M1-related cytokine) was decreased (Additional file 1: Figure S1C). These results suggested that *Cs*GRN could induce the polarization of M2 macrophages in the mouse liver, possibly as a result of an interaction between macrophages and HIBECs treated with *Cs*GRN.

### *Cs*GRN-induced cell–cell interaction promoted malignant transformation of HIBECs

**Fig. 4:**
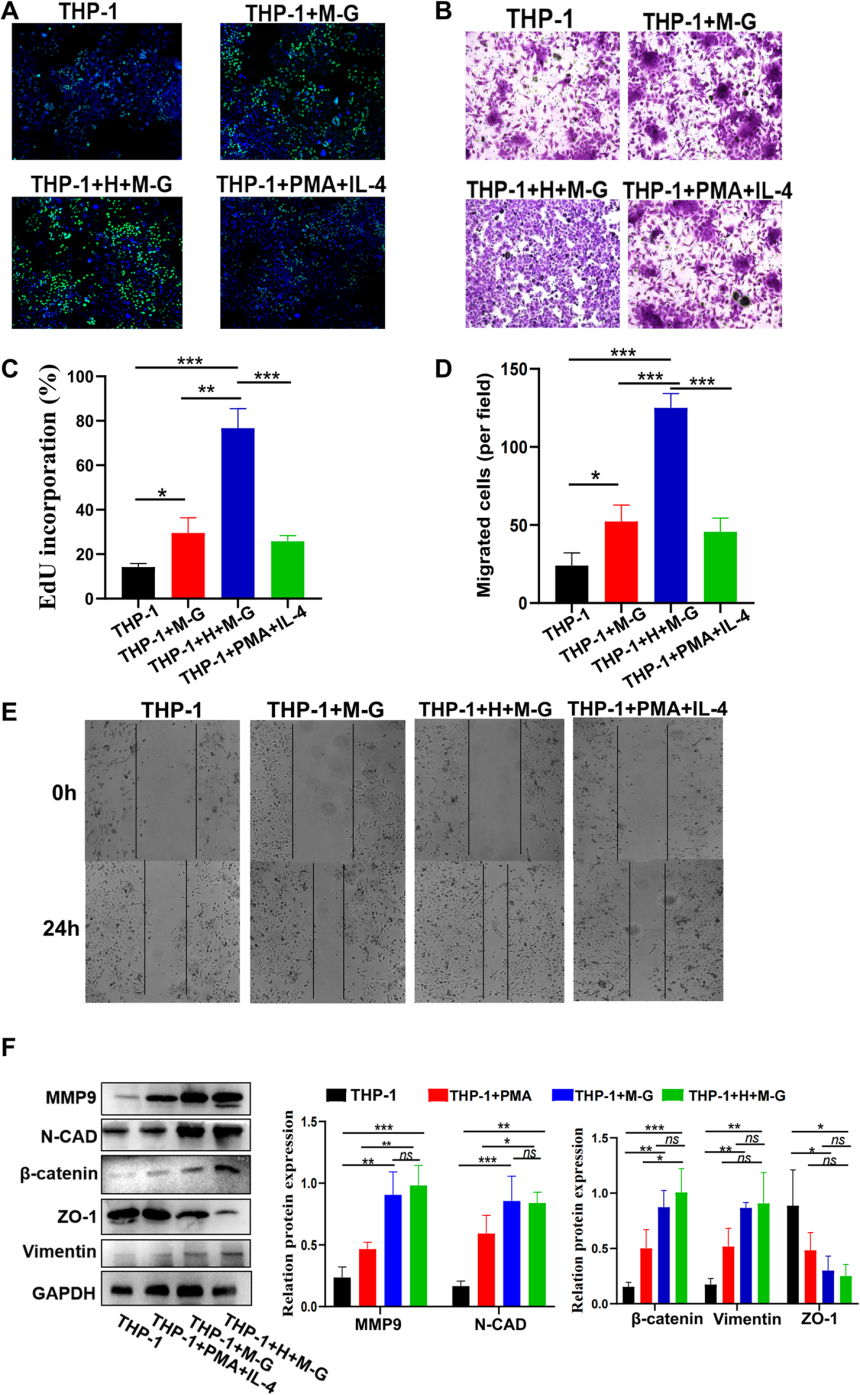
**a**–**f** *Cs*GRN-induced interaction between HIBECs and macrophages promoted malignant transformation of HIBECs. **a**, **c** The proliferation of HIBECs in the co-culture system was assessed by EdU-488 assay. Scale bar 10 μM. Transwell assays (**b**,** d**) and a wound healing assay (**e**) were used to estimate the migration capacity of HIBECs in the co-culture system. **f** Western blot was used to determine the expression level of EMT-related proteins of HIBECs in the co-culture system. *ns* Not significant, * *P* < 0.05, ** *P* < 0.01, **** P < 0.0001. For other abbreviations, see Figs. 1, 2 and  3

Next, we wanted to explore if macrophages were necessary for *Cs*GRN to induce malignant transformation of HIBECs. Hence, we constructed a co-culture system of THP-1 cells (upper chamber) with HIBECs (lower chamber), which were cultured with or without supernatant from HIBECs (Additional file 1: Figure S1B). THP-1-PMA/IL-4 was also used as a positive control. We found that both THP-1-HIBECs-MBP-*Cs*GRN and THP-1-MBP-*Cs*GRN groups comprised a higher proportion of EdU-positive cells (stained green) (Fig. 4a, c), indicating that *Cs*GRN-activated M2-type macrophages could induce the malignant proliferation of HIBECs through cell–cell interaction. Moreover, Transwell cell migration and wound healing assays showed that HIBECs exhibited elevated migration capacities in the THP-1-HIBECs-MBP-*Cs*GRN group compared to the controls (Fig. 4b, d, e). Additionally, western blot showed that matrix metalloproteinase 9, N-cadherin, vimentin, and β-catenin were increased and ZO-1 was decreased in HIBECs of THP-1-PMA/IL-4, THP-1-MBP-*Cs*GRN and THP-1-HIBECs-MBP-*Cs*GRN, which revealed the EMT of HIBECs in these groups (Fig. 4f). These results indicated that *Cs*GRN-induced interactions between M2 macrophages and HIBECs promoted aberrant, malignant transformation of HIBECs.

### *Cs*GRN-induced cell–cell interactions in biliary epithelial cells through IL-6/JAK2/STAT3 pathways

**Fig. 5:**
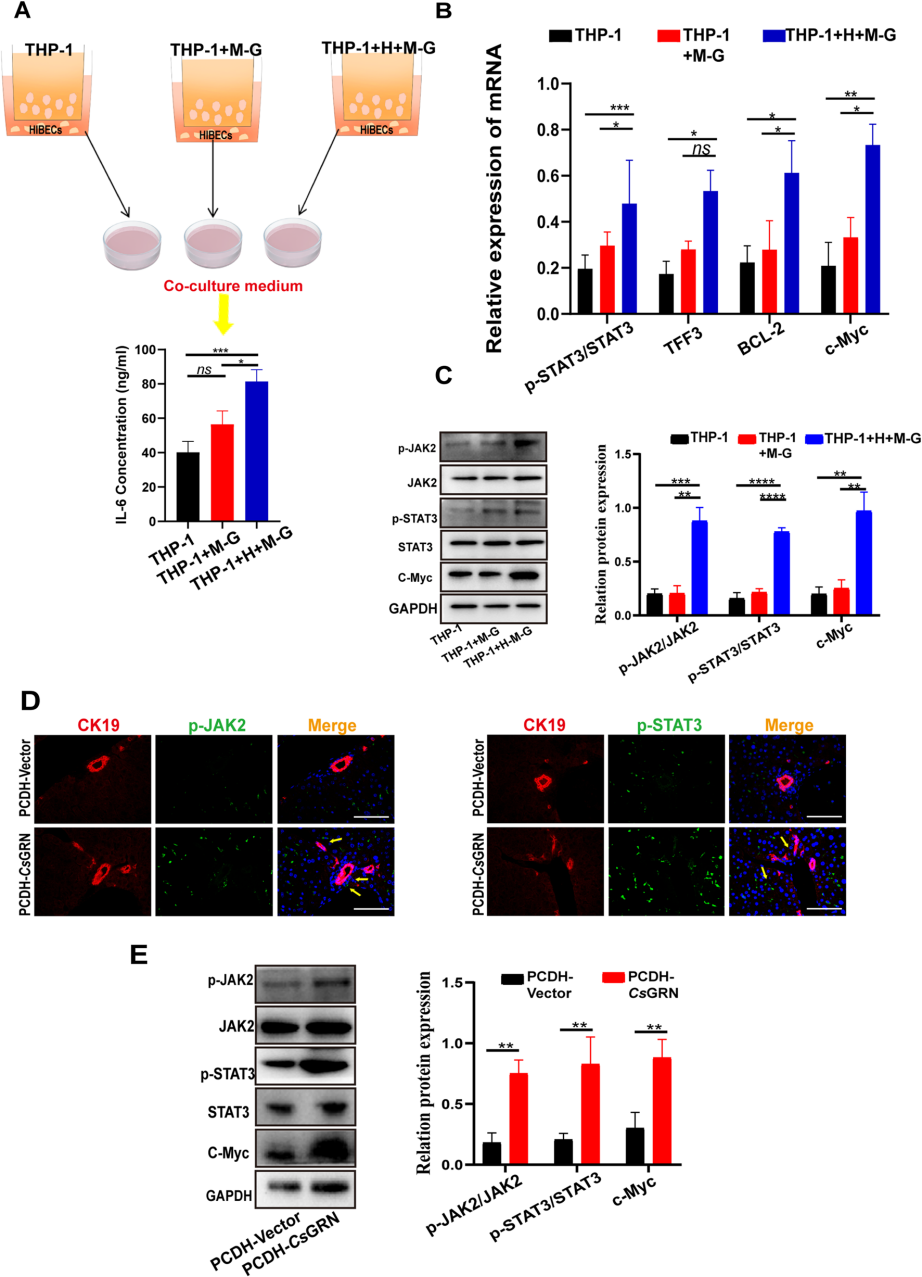
**a**–**e** IL-6/Janus kinase 2 (JAK2)/STAT3 signaling pathway was involved in the cell–cell interaction mediated by *Cs*GRN activated in HIBECs. **a** ELISA was used to determine the concentration of IL-6 in the co-culture medium*.* **b** Expression of phosphorylatedSTAT3 (*p-STAT3*), TFF3, BCL-2 and c-Myc in HIBECs treated with co-culture medium was detected by qPCR. **C** Western blot analysis of p-JAK2, p-STAT3 and c-Myc levels in the co-culture medium. **d** Expression of p-JAK2+ CK19+ cells and p-STAT3+ CK19+ cells in the liver of *Cs*GRN-injected mice was assessed by IF. Scale bar 100 μM.** e** Western blot analysis of p-JAK2, p-STAT3 and c-Myc levels in the liver of *Cs*GRN-treated mice. * *P* < 0.05, ** *P* < 0.01, *** P < 0.001, **** *P* < 0.0001. For other abbreviations, see Figs. 1, 2,  3 and  4

Previous studies have reported that IL-6, an inflammatory cytokine produced in the liver, plays an important role in promoting transformed cell proliferation in human malignant cholangiocytes [20]. Accordingly, we supposed that IL-6 could mediate the interaction between M2- macrophages and HIBECs. The ELISA assay results showed that the THP-1-MBP-*Cs*GRN and THP-1-HIBECs-MBP-*Cs*GRN groups secreted more IL-6 than the THP-1 group (Fig. 5a). Next, we found that increased IL-6 levels led to higher p-JAK2 and p-STAT3 levels in HIBECs co-cultured with the THP-1-HIBECs-MBP-*Cs*GRN group, which led to the increased expression of downstream-intiated proteins c-Myc, TFF3 and Bcl-2 (Fig. 5b, c). Levels of p-JAK2 and p-STAT3 in the liver tissue were detected by IF and western blot. The IF results showed that the CK19+ p-JAK2+ cells and CK19+ p-STAT3+ cells in *Cs*GRN-treated livers were also significantly increased, which was in accordance with the in vitro results (Fig. 5d, e). These findings indicated that *Cs*GRN can activate the JAK2/STAT3 pathway and then regulate the synthesis of downstream proteins, and thus contribute to malignant transformation of HIBEC.

### Malignant transformation of HIBECs induced by *Cs*GRN was associated with MEK/ERK pathway activation and STAT3 phosphorylation

**Fig. 6:**
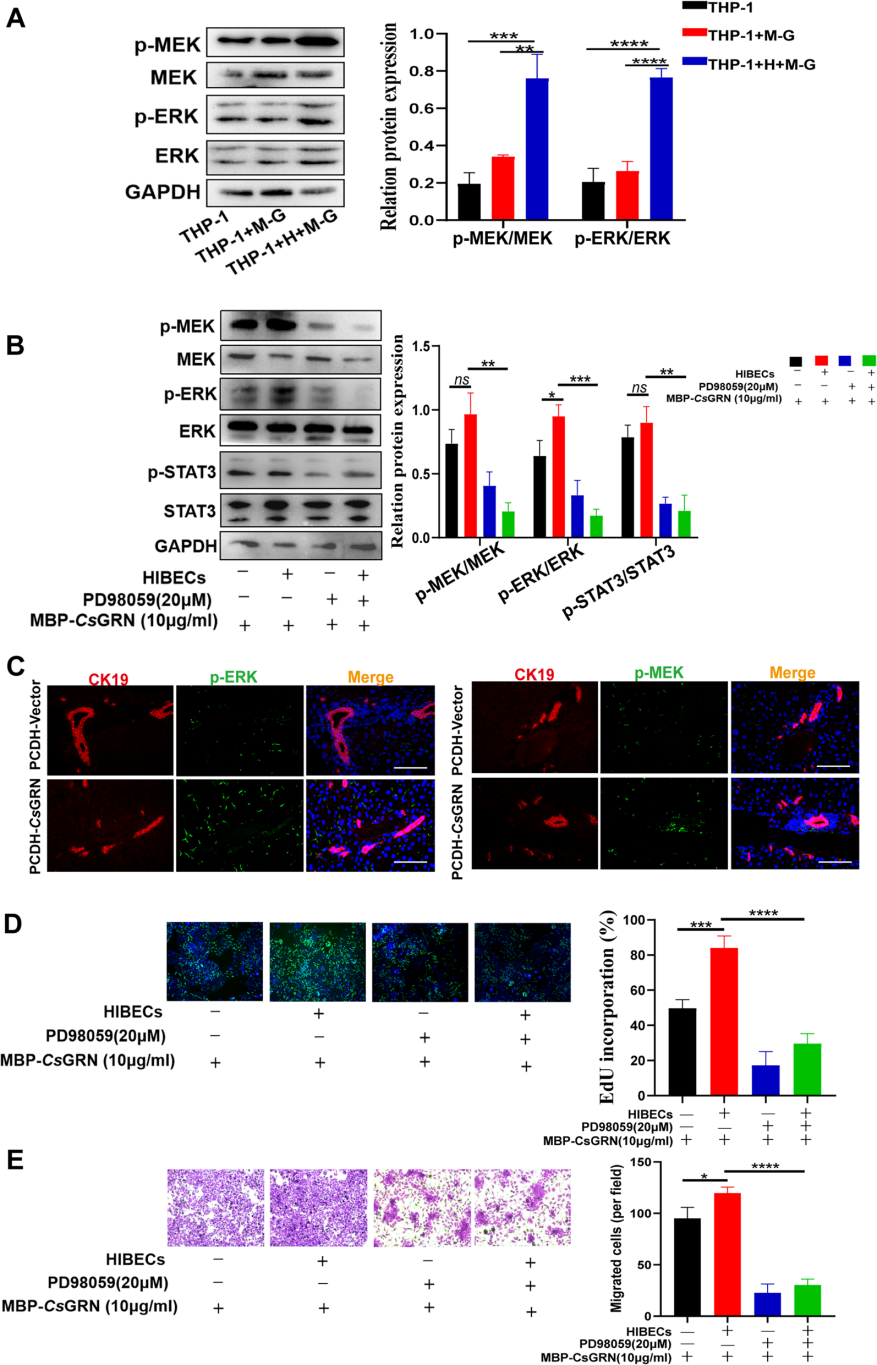
**a**–**e** Mitogen-activated protein kinase (MEK)/extracellular signal-regulated kinase (ERK) was involved in the malignant proliferation and metastasis of HIBECs caused by *Cs*GRN-mediated cell–cell interaction. **a** The expression of phosphorylated (p-) MEK (p-MEK) and p-ERK in the co-culture medium (Fig. 5) and with MEK/ERK inhibitor co-culture medium (**b**) were assessed by western blot. **c** The expression of p-MEK+ CK19+ cells and p-ERK+ CK19+ cells in the liver of *Cs*GRN-injected mice were assessed by IF. Scale bar 100 μM. EdU-488 assay (**d**) and Transwell assay (**e**) were performed to evaluate the migration or proliferation of HIBECs after treatment with MEK/ERK inhibitor. Scale bar 10 μM. * *P* < 0.05, ** *P* < 0.01, *** P < 0.001, **** *P* < 0.0001. For other abbreviations, see Figs. 1, 2 and  3 and 4

IL-6 has been proved to regulate cation-selective epithelial tight junction permeability through aberrant activation of the MEK/ERK pathway [21]. Western blot and IF were used to determine the protein expression level of p-MEK and p-ERK with or without phosphorylation. p-MEK and p-ERK were only significantly increased in THP-1-HIBECs-MBP-*Cs*GRN groups and *Cs*GRN-injected mice (Fig. 6a, c). To confirm that *Cs*GRN-mediated cell interactions are regulated through the MEK/ERK pathway, we conducted inhibition experiments by adding 20 mM of PD98059 (an inhibitor of the MEK/ERK pathway) during the co-culture (Additional file 1: Figure S1E). The inhibitor treatment markedly decreased the expression of p-MEK, p-ERK and p-STAT3 (Fig. 6b). In addition, the malignant proliferation and metastasis of HIBECs in the THP-1-HIBECs-MBP-*Cs*GRN group were strongly abrogated after co-culturing with MEK/ERK pathway inhibitor (Fig. 6d, e). To summarize, our result indicated that the MEK/ERK pathway was involved in *Cs*GRN-induced STAT3 phosphorylation and further affected malignant transformation of HIBECs.

## Discussion

Chronic infection with *C. sinensis* can cause hepatobiliary aberrations, which may lead to the abnormal proliferation of cholangiocytes through the disruption of redox homeostasis and dysregulation of physiological signaling pathways [22]. It is believed that CCA carcinogenesis is a multi-step process that entails the recognition of normal cholangiocytes by the pathogen, chronic inflammation, wound healing, proliferation of cells, genetic and epigenetic mutations, and malignant transformation of cholangiocytes through a series of subsequent events [3]. It has been reported that *Cs*ESPs are responsible for activating multiple oncogenic pathways [23]. In addition, Toll-like receptors activated by *Cs*ESPs can enzymatically generate free radicals, which in turn promote nuclear factor kappa B–mediated inflammatory processes [24]. In earlier studies, we found that *Cs*GRN, an important component of *Cs*ESPs, is involved in the process of malignant transformation of hepatocytes as well as the metastasis of HCC and CCA cells in vitro [14]. However, it is still unclear if *Cs*GRN is also involved in the immunopathogenesis of cholangiocarcinogenesis following *C. sinensis* infection. In the present study, we found that *Cs*GRN can facilitate abnormal proliferation and metastasis of biliary epithelial cells by inducing EMT, which plays an essential role in carcinogenesis, wound healing, and organ fibrosis [25, 26]. Moreover, we also demonstrated that *Cs*GRN causes biliary damage by initiating inflammation around the bile duct in the mouse liver. These reactions, which are called ductular reactions [27], result in abnormal proliferation of cholangiocytes in the inflammatory microenvironment of intrahepatic biliary neoplasia [28]. We also found that the level of ductular reaction marker CK19 was increased in the bile duct of *Cs*GRN-treated mice. Therefore, our results indicate that *Cs*GRN significantly contributes to the malignant transformation of HIBECs in vitro and biliary injuries in vivo, and demonstrate that *Cs*GRN could be a cancerogenic factor in *Clonorchis sinensis*-induced CCA.

In addition to fighting infections with worms and inducing hyperimmune responses, macrophages play a major role in regulating these immune responses via the release of inflammatory mediators and cytokines, which can also result in bile duct hyperplasia [29]. In the *Cs*GRN-treated mice livers, macrophages and other inflammatory cells were markedly increased and more IL-6 and MCP-1 were secreted. MCP-1 is a pro-inflammatory chemokine released by liver cells, such as biliary epithelial cells, stellate cells, and hepatocytes. It plays an important role in mobilizing mononuclear cells [30]. We found that the production of MCP-1 protein was dramatically increased in HIBECs treated with *Cs*GRN. The bile duct tissue of mice treated with *Cs*GRN also showed an increase in MCP-1 levels in the peribiliary region. Due to the regulatory effect of MCP-1 on macrophage infiltration, we hypothesized that *Cs*GRN stimulates the expression of MCP-1 in HIBECs, leading to the recruitment of macrophages and the differentiation of M2-type macrophages. Therefore, we co-cultured recombinant *Cs*GRN protein with THP-1 cells, and the cells that showed the M2 phenotype had increased expression of CD206 compared with control groups. To further determine the role of macrophages in *Cs*GRN-regulated malignant transformation of biliary epithelial cells, we designed a co-culture system of THP-1 cells and HIBECs to mimic the *Cs*GRN-treated microenvironment. We found that cell interactions between macrophages and HIBECs was necessary for *Cs*GRN to induce malignant transformation of HIBECs.

The IL-6/STAT3 pathway is abnormally activated in HCC, which results in increased progression, invasion, and migration of the cancer cells [31]. Activation of STAT3 is an important hallmark as it contributes to the initiation and progression of cancer, which enhances cell proliferation, migration, invasion, and angiogenesis through transcriptional regulation. In addition, through the activation of STAT3 and upregulation of downstream TFF3, IL-6 facilitates the migration of HIBECs [32–35]. Here, we found that IL-6 significantly activated JAK2/STAT3 signaling in the co-culture system of HIBECs and THP-1 cells treated with *Cs*GRN. The expression levels of p-JAK and p-STAT3 were significantly enhanced around the intrahepatic bile duct regions. Additionally, we found that STAT3 proteins mediated anti-apoptotic activity by upregulating c-Myc, BCL-2, and TFF3 genes, which resulted in hyperplasia and the migration of HIBECs.

In addition to activating JAK2, IL-6 can also activate the phosphorylation of STAT3 via the MEK/ERK pathway [36]. IL-6 can regulate epithelial tight junction permeability by activating the MEK/ERK and PI3K/AKT pathways [21]. Moreover, IL-6 canonical signaling can stimulate the transient phosphorylation of STAT3, and the trans-signaling pathway can lead to activation of MEK signaling [37]. We found that the MEK signaling pathway was upregulated in THP-1 cells cultured with the supernatant of *Cs*GRN-treated HIBECs, and played a crucial role in malignant transformation of HIBECs. Meanwhile, we also found that the *Cs*GRN-injected mouse model exhibited higher expression of p-MEK and p-ERK in the bile ducts of the liver than the control group. Additionally, blocking the MEK/ERK pathway with PD98059 (an inhibitor of the MEK/ERK pathway) inhibited the abnormal proliferation and metastasis of HIBECs, and decreased the expression level of p-STAT3. It is well known that activation of the JAK/STAT3 signaling pathway results in STAT3 dimerization, and that the dimers translocate to the nucleus and are involved in inducing the expression of pivotal genes [38]. The results of the present study indicated that *Cs*GRN stimulated HIBECs co-cultured with macrophages to secrete IL-6, which activated the JAK2 and MEK/ERK pathway, leading to the elevation of the expression of p-STAT3, which resulted in the malignant transformation of HIBECs.

## Conclusions

The results indicated that M2-type macrophages were recruited during the malignant transformation of biliary epithelial cells that was promoted by *Cs*GRN. Moreover, interactions between HIBECs and M2 macrophages caused a significant increase in the level of IL-6. Activation of the JAK2/STAT3 and MEK/ERK pathways by HIBECs may regulate STAT3 phosphorylation, which promotes excessive hyperplasia and abnormal proliferation. All of these mechanisms may ultimately result in cholangiocarcinoma.

## Supplementary Information

**Additional file 1: Figure S1. A **After treatment with 10μg/ml of recombinant MBP-*Cs*GRN proteins or MBP proteins in HIBEC cells for 24 h, the expression of MCP-1, IL-6, and COX-2 proteins was detected by western blot.* NC* blank group. Treatment with MBP proteins was used as the negative control. ** *P* < 0.01, *** P < 0.001 **B **Transwell assay to estimate invasion of THP-1 cells in the co-culture system. *ns* Not significant, ** P < 0.001** C** Expression of Arg1 and iNOS in the migrated THP-1 cells was detected by qPCR. (first line). *ns* Not significant, *** P < 0.001. The expression of IL-6 and IL-10 secreted by migrated THP-1 cells were examined by ELISA (second panel). *ns* Not significant, * *P* < 0.05, ** *P* < 0.01. **D** Cell co-culture system for *Cs*GRN-treated HIBECs and THP-1cells. THP-1+PMA+IL-4 treatment as the positive control.** E** MEK/ERK inhibitor PD98059 (20 μM) was added to the co-culture medium.

## Data Availability

Supporting data for the conclusions of this article are included within the article. The raw data supporting the conclusions of this article will be made available by the authors without undue reservation.

## Abbreviations

CCA: Cholangiocarcinoma
*Cs*GRN: *Clonorchis sinensis* granulin
ELISA: Enzyme-linked immunosorbent assay
EMT: Epithelial-to-mesenchymal transition
ERK: Extracellular signal-regulated kinase
ESPs: Excretory/secretory products
HCC: Hepatocellular carcinoma
HIBECs: Human intrahepatic biliary epithelial cells
IF: Immunofluorescence
IHC: Immunohistochemistry
IL: Interleukin
JAK: Janus kinase
MEK: Mitogen-activated protein kinase
PCR: Polymerase chain reaction
STAT3: Signal transducer and activator of transcription 3
