# Supplementary figures and images for: Clonorchis sinensis granulin promotes malignant transformation of human intrahepatic biliary epithelial cells through interaction with M2 macrophages via regulation of STAT3 phosphorylation and the MEK/ERK pathway
Source: Parasit Vectors. 2023 Apr 24;16:139. doi: 10.1186/s13071-023-05765-6 (PMC10124682; doi:10.1186/s13071-023-05765-6)

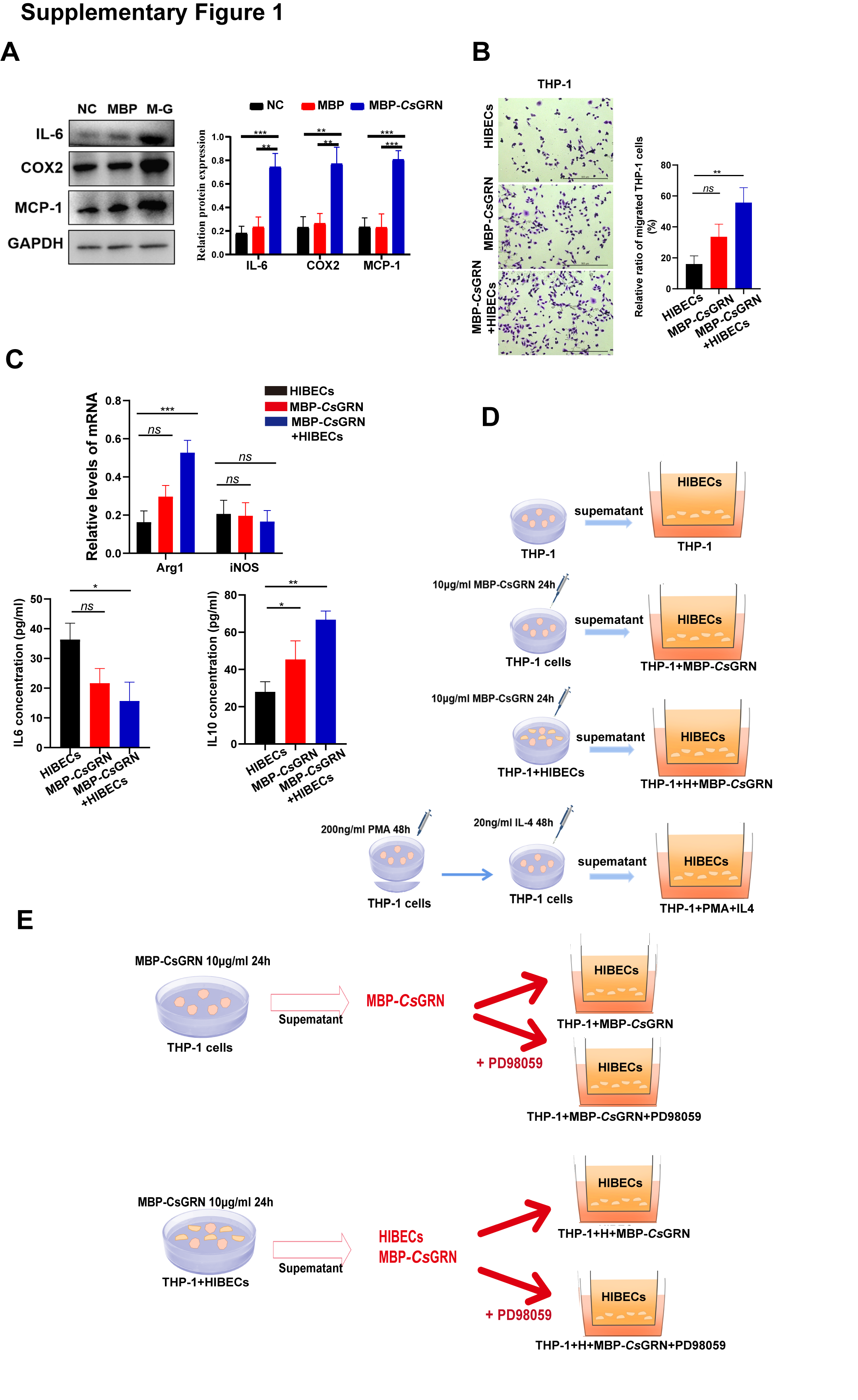

Supplement: Supplementary file 1 — Additional file 1: Figure S1. A After treatment with 10μg/ml of recombinant MBP-CsGRN proteins or MBP proteins in HIBEC cells for 24 h, the expression of MCP-1, IL-6, and COX-2 proteins was detected by western blot. NC blank group. Treatment with MBP proteins was used as the negative control. ** P < 0.01, *** P < 0.001 B Transwell assay to estimate invasion of THP-1 cells in the co-culture system. ns Not significant, ** P < 0.001 C Expression of Arg1 and iNOS in the migrated THP-1 cells was detected by qPCR. (first line). ns Not significant, *** P < 0.001. The expression of IL-6 and IL-10 secreted by migrated THP-1 cells were examined by ELISA (second panel). ns Not significant, * P < 0.05, ** P < 0.01. D Cell co-culture system for CsGRN-treated HIBECs and THP-1cells. THP-1+PMA+IL-4 treatment as the positive control. E MEK/ERK inhibitor PD98059 (20 μM) was added to the co-culture medium. [file 13071_2023_5765_MOESM1_ESM.tif]
